# Metamorphosis of Topical Semisolid Products—Understanding the Role of Rheological Properties in Drug Permeation under the “in Use” Condition

**DOI:** 10.3390/pharmaceutics15061707

**Published:** 2023-06-11

**Authors:** Xuping Jin, Seyed Ebrahim Alavi, Abbas Shafiee, Vania Rodrigues Leite-Silva, Kiarash Khosrotehrani, Yousuf Mohammed

**Affiliations:** 1 Frazer Institute, Faculty of Medicine, The University of Queensland, Brisbane, QLD 4102, Australia; xuping.jin@uqconnect.edu.au (X.J.); s.ebrahimalavi@uq.edu.au (S.E.A.); a.shafiee@uq.edu.au (A.S.); v.rodriguesleiteesilva@uq.edu.au (V.R.L.-S.); k.khosrotehrani@uq.edu.au (K.K.); 2 School of Pharmacy, The University of Queensland, Brisbane, QLD 4102, Australia; 3 Instituto de Ciências Ambientais, Químicas e Farmacêuticas, Departamento de Ciências Farmacêuticas, Universidade Federal de São Paulo, UNIFESP, Diadema 09913-030, Brazil

**Keywords:** metamorphosis, topical semisolid products, lidocaine, rheological properties, in vitro permeation studies

## Abstract

When developing topical semisolid products, it is crucial to consider the metamorphosis of the formulation under the “in use” condition. Numerous critical quality characteristics, including rheological properties, thermodynamic activity, particle size, globule size, and the rate/extent of drug release/permeation, can be altered during this process. This study aimed to use lidocaine as a model drug to establish a connection between the evaporation and change of rheological properties and the permeation of active pharmaceutical ingredients (APIs) in topical semisolid products under the “in use” condition. The evaporation rate of the lidocaine cream formulation was calculated by measuring the weight loss and heat flow of the sample using DSC/TGA. Changes in rheological properties due to metamorphosis were assessed and predicted using the Carreau–Yasuda model. The impact of solvent evaporation on a drug’s permeability was studied by in vitro permeation testing (IVPT) using occluded and unconcluded cells. Overall, it was found that the viscosity and elastic modulus of prepared lidocaine cream gradually increased with the time of evaporation as a result of the aggregation of carbopol micelles and the crystallization of API after application. Compared to occluded cells, the permeability of lidocaine for formulation F1 (2.5% lidocaine) in unoccluded cells decreased by 32.4%. This was believed to be the result of increasing viscosity and crystallization of lidocaine instead of depletion of API from the applied dose, which was confirmed by formulation F2 with a higher content of API (5% lidocaine) showing a similar pattern, i.e., a 49.7% reduction of permeability after 4 h of study. To the best of our knowledge, this is the first study to simultaneously demonstrate the rheological change of a topical semisolid formulation during volatile solvent evaporation, resulting in a concurrent decrease in the permeability of API, which provides mathematical modelers with the necessary background to build complex models that incorporate evaporation, viscosity, and drug permeation in the simulation once at a time.

## 1. Introduction

Generally, most topical products are produced in semisolid dosage forms, such as creams, ointments, gels, lotions, and emulsions [1], and most of them target the skin or subcutaneous tissue [2]. These products must increase the permeation of drug molecules and preserve the rate and extent of penetration properly in the skin layers to produce appropriate therapeutic effects [1,2,3]. Different factors, including the physicochemical properties of the active pharmaceutical ingredient (API), the interaction between formulation excipients and the skin, drug interaction with the skin, drug interaction with the excipients, and an overall interplay of the skin, API, and excipients, determine the therapeutic effects of topical drugs [4].

The US Food and Drug Administration (FDA) has recently released product-specific guidance in order to fabricate topical drugs with in vitro options consisting of qualitative sameness (Q1) and quantitative sameness (Q2) assessment of formulations, physiochemical and structural characterization of formulations (Q3), and in vitro evaluation of the drug release and permeation of the formulations [5]. These tests evaluate the critical quality attributes (CQAs; e.g., rheological properties, thermodynamic activity, ionization, particle and globule size, and the rate/extent of drug release/permeation) of the topical semisolid products to ensure the desired quality of the products [1,6,7,8,9]. However, these CQAs of the drug products can be changed under “in use” conditions due to various metamorphotic events that can ultimately cause specific failure modes for the products [10,11,12].

The metamorphosis of topical pharmaceutical products refers to the changes or transformations that occur in a topical product over time, which include alterations in the physical, chemical, or pharmaceutical properties of the product. During their shelf life or upon application to the skin, these products can undergo various metamorphic changes. For instance, physical metamorphosis may involve changes in the consistency, rheology, or texture of the product. Previous literature [13] already showed that a cream dosed with a pump may be subjected to higher shear compared to the formulation dosed with another technique, resulting in different drop sizes on skin. These changes can impact the product’s bioavailability and therapeutic effect and eventually lead to product failure. An example of a potential failure mode is when the buffering capacity of the skin alters the pH of the formulation, thereby affecting the solubility of API and subsequently influencing the concentration gradient, i.e., the thermodynamic activity, which serves as the driving force for permeation. Factors that can contribute to the metamorphosis of topical pharmaceutical products include temperature, humidity, exposure to light, interactions between ingredients, the stability of APIs, etc. Higher temperatures can increase the possibility of degradation, while the moisture in a high-humidity environment is likely to affect the stability of the formulation by causing phase separation. To mitigate such impacts and failure modes, metamorphosis can be controlled from formulation development through process parameters and the quality target product profile (QTPP). Manufacturers develop a final QTPP and manufacturing process throughout the development process with measures, such as the use of controlled temperature and pressure conditions, that are built into the process [14,15]. The metamorphosis of formulation can also be modulated by using excipients. Viscosity enhancers, which are commonly added in formulations to modify the viscosity of products, also reduce the evaporation rate of volatile solvents due to their increased viscosity.

Under “in use” conditions, a limited amount of formulation is administered on the skin surface, where the physicochemical properties of the formulation can be significantly changed from a primary state, referring to the deposition of the original formulation in the primary container; to a secondary state, referring to the administered formulation during the dynamic stage of losing volatile vehicle ingredients; and finally to a tertiary state, referring to the final formulation, where all volatile ingredients become evaporated from the administered product on top of the skin [16]. During this metamorphosis, a series of events that can change the microstructure of topical formulations, such as supersaturation and crystallization, can change the permeation profile of APIs as well as drug solubility and thermodynamic activity and further lead to specific failure modes for topical semi-solid products [17,18,19]. Previous literature [20,21,22] has shown the relationship between the metamorphosis, physicochemical properties, and permeation of APIs. Coldman et al. [20] evaluated the human skin penetration of ^14^C-labeled fluminolone acetonide and its acetate ester in vitro at 37 °C. In this study, the vehicle mixtures of isopropanol and isopropyl myristate or propylene glycol were examined, and the results demonstrated an 8- to 10-fold higher penetration of fluocinolone acetonide in the formulations containing isopropanol compared to the formulations containing non-volatile vehicles, which was attributed to the supersaturation of fluocinolone in the formulation resulted from the evaporation of isopropanol [20]. Chia-Ming et al. [21] performed two sets of skin permeation experiments in vitro to evaluate topical minoxidil delivery and the role of thermodynamic activity. In this study, minoxidil at different concentrations (0.5%. l%, 2%, 3%. 4%, and 5%) in a hydroalcoholic vehicle (fixed composition of propylene glycol/water/ethanol (20.0:63.2:16.8) was prepared, and the results showed a reduction in the flux of minoxidil at different concentrations (3%, 4%, and 5%) in formulations composed of propylene glycol, ethanol, and water. The decrease in flux was due to the crystallization of minoxidil after the evaporation of volatile vehicles. To predict the “in use” penetration profile of metronidazole semisolid products under clinical conditions, Arora et al. [23] developed a physiologically-based pharmacokinetic model of metronidazole using in vitro permeation testing (IVPT) data, which successfully captured the metamorphosis of metronidazole gel and cream after application. However, despite its relevance for the development of topical semisolid products, there are only limited attempts to correlate the changes in the rheological properties of formulation during metamorphosis with the permeation of API.

To our knowledge, the metamorphosis of topical pharmaceutical products, especially under “in use” conditions where a product is finitely thin-layer dosed in a dermatologically relevant environment, is understudied. This study aimed to use lidocaine as a model drug to demonstrate the connection between evaporation and simultaneously changing rheological properties and permeation of APIs in topical semisolid products under the “in use” condition. Lidocaine, as a hydrophobic drug, suffers from poor solubility in topical anestric products. With the evaporation of solvents, they tend to crystallize rapidly because of their altered solubility. Therefore, the evaporation rate of prepared eutectic oil (O)/water (W) creams of lidocaine was evaluated using a thermal analysis system, rheological properties were monitored by a rheometer, and the permeability was studied with comparative IVPT by controlling the evaporation condition of the formulation. Determining the metamorphotic events helps to understand their influence on the in vitro permeation profile and mitigate the potential failure mode of topical semisolid products. Along with these physicochemical characterizations, the revealed underlying mechanism provides mathematical modelers with the necessary background to build complex models that incorporate evaporation, viscosity, and drug permeation in the simulation once at a time.

## 2. Materials and Methods

### 2.1. Chemicals

Lidocaine USP and prilocaine USP were purchased from Spectrum Chemical Mfg. Corp. (Gymea, NSW, Australia). Phosphate-buffered saline (PBS) sachets (pH 7.4), isopropyl alcohol (IPA), sodium azide, isopropyl myristate (IPM), sodium lauryl sulfate (SLS), acetonitrile, sodium hydroxide, ethanol, di-sodium hydrogen phosphate, and sodium phosphate monobasic of analytical grade were purchased from Sigma-Aldrich (Macquarie Park, NSW, Australia). Carbopol 980 and Volpo™ N20 were prepared by Lubrizol Corporation (Silverwater, NSW, Australia) and Croda (Parramatta, NSW, Australia), respectively. Milli-Q water was used in this work, unless otherwise noted.

### 2.2. Preparation of Formulations

Due to the poor solubility and temperature sensitivity, the optimized lidocaine: IPA/water eutectic mixture was utilized to prepare the cream formulation, where the lidocaine was melted at room temperature to form the oil phase, based on previous literature [24]. In the preparation of the lidocaine O/W cream, materials listed in Table 1 were weighted and then homogenized at 20,000 RPM for 10 min at 25 °C using ULTRA-TURRAX^®^ T18 (IKA, UK) before being stored in air-tight plastic containers. Sodium lauryl sulfate and carbopol 980 were used as the surfactant and thickening agent, respectively.

As shown in Table 2, a series of carbopol fluids were prepared with concentrations ranging from 0.1% to 1.0% (*w*/*w*). The pH of carbopol fluids was adjusted to 7 by neutralizing carbopol 980 with a 5.39 M sodium hydroxide solution in a weight ratio of 2.3:1. The prepared fluids were degassed for 5 min with an ultrasound water bath before being stored in airtight containers.

### 2.3. Determination of Weight Loss by Evaporation

The evaporation rate of lidocaine cream samples was assessed using a thermal analysis system equipped with thermogravimetric analysis (TGA) and differential scanning calorimetry (DSC) analyses (TGA/DSC3+, Mettler Toledo, Columbus, OH, USA). Five milligrams of cream samples were weighed in alumina crucibles for measurement. Samples were held isothermally at the experimental temperature of 32 °C for 240 min. The weight of the sample and heat flow were then measured, and weight percentage change versus time and heat flow versus time were plotted. The evaporation rate was then determined and plotted versus time by calculating the actual weight loss per unit time using the equation published elsewhere [25].

### 2.4. Measurement and Modeling of ‘in Use’ Apparent Viscosity

The apparent viscosity of lidocaine cream and carbopol gel samples was measured as a function of shear rate from 0.001 to 10,000 s^−1^ using a controlled shear rate sweep test with an MCR 302e rheometer (Anton Paar, North Ryde, NSW, Australia). A sufficient volume of formulations was carefully loaded onto the stage to form a homogeneous and thin layer of samples before the parallel plate geometry of 40 mm diameter moved to a measuring gap of 0.5 mm. The protruded sample was then trimmed before covering an alloy hood on the stage to mitigate the wall-slipping effect at high velocity. The temperature of the sample was equilibrated at 32 ± 0.5 °C for 2 min using a P-PTD200 measuring cell to simulate the surface temperature of the skin before measurements. Specifically, to understand the change of viscosity under the “in use” condition, a fresh thin layer of lidocaine cream samples was left open on the stage each time at this temperature to dry, and the evaporation state of the samples was controlled by the time for drying, i.e., 0, 15, 30, 45, 60, 75, 90, 105, and 120 min. Thereby, the apparent viscosity was obtained at various evaporation states. The polymer concentration in a formulation is subjected to the change in volatile solvent amount with evaporation, which further leads to changes in viscosity [25]. Thereby, the viscosity of carbopol liquid at various concentrations was studied in this work to understand the influence of continuously changing carbopol concentration on rheological properties so that this kind of impact can be estimated in an evaporating formulation. The studies were performed in a temperature- and humidity-controlled facility where ambient temperature and humidity were closely monitored because the metamorphosis can be impacted by environmental factors. All measurements were taken in triplicate using fresh samples. Retrieved data is fitted to the Carreau–Yasuda model (Equation (1)):

(1) $$\eta_{a}=\eta_{\infty }+\left(\eta_{0}-\eta_{\infty }\right){\left[1+{\left(\lambda \dot{\gamma }\right)}^{a}\right]}^{\frac{n-1}{a}},$$

where $\dot{\gamma }$ is the shear rate (s^−1^), $\eta_{a}$ is the apparent viscosity (Pa·s), $\eta_{\infty }$ is the infinite shear viscosity (Pa·s), $\eta_{0}$ is the zero-shear viscosity (Pa·s), λ is the time constant (s), a is the transition control factor, and n is the power index. Additionally, the water activity of CBP01–CBP10 was measured using an Aqualab Pawkit water activity meter (METER Group Inc., Pullman, WA, USA). In brief, 3 mL of samples were placed homogeneously in measuring chamber and measured in triplicates after calibration with the provided standard solutions.

### 2.5. Determination of Viscoelastic Properties

The viscoelastic properties of lidocaine cream samples, including storage modulus, loss modulus, and complex viscosity, were measured with the condition and sample treatment procedures as described in the preceding section. To identify the linear viscoelastic region (LVR) of samples, dynamic strain sweep tests were performed by increasing % strain logarithmically from 0.01% to 1000% at a frequency of 6.28 rad/s and a temperature of 32 ± 0.5 °C. The measurements were taken at 10 points per decade in log mode. After the determination of LVR, a constant deformation of 1% strain was selected for frequency sweep tests over a range of 0.1–100 rad s^−1^ at 32 ± 0.5 °C to interrogate the oscillatory rheogram of lidocaine cream samples before and after 2-h evaporation. The measurements were taken at 5 points per decade in log mode. All measurements were taken in triplicate using fresh samples.

### 2.6. In Vitro Permeation Test (IVPT)

Full-thickness skin was immediately defatted after excision from the abdominal area of 25- to 48-year-old female patients undergoing plastic surgery with approval by Metro South and the University of Queensland Human Research Ethics Committee (2018/HE001721). The epidermis was heat separated using pre-established procedures before being stored at −40 °C until use [26].

For the permeation study, heat-separated epidermis membranes were sandwiched between donor chambers and receptor chambers of Franz diffusion cells set in a circulated water bath maintained at 37 ± 0.5 °C. The leakage test and skin impedance test were performed using a standard digital multimeter (FINEST 500) at 20 kΩ to exclude any impaired epidermis membranes prior to the study. After the equilibration of diffusion cells and receptor medium for 30 min, 110 mg of prepared lidocaine cream samples were dosed onto the membrane with an exposure area of 1.13 cm^2^. PBS at pH = 7.4 with 0.5% (*w*/*w*) Volpo™ N20 and 0.05% (*w*/*w*) sodium azide was selected as the receptor medium, which was continuously stirred by a magnetic stir minibar placed in the receptor chamber at 600 RPM to maintain the sink condition. The evaporation condition of the formulation was controlled by the occlusion of the donor compartment. The evaporation was mitigated in occluded cells by covering the donor chamber with Parafilm, while unoccluded cells were left open to the environment. The receptor medium of 3.2 mL was fully collected from the receptor chamber and replaced with fresh medium at 0.5, 1, 1.5, 2, 3, 4, 5, 6, 7, and 8 h. The study was performed in triplicate.

After the study, 50 µL of the collected receptor medium was spiked into 50 µL of internal standard solution (5 µg/mL prilocaine) and vortexed for HPLC analysis using a Shimadzu Prominence system with a SIL20-AHT autosampler, an SPD20A detector set to 210 nm, and a PSC18-100 A° column with temperature maintained at 35 °C. 20 µL of this prepared sample was eluted under isocratic flow with a mobile phase consisting of acetonitrile (0.23 mL/min) and 0.5 M sodium phosphate buffer at pH = 5.8 (0.73 mL/min). The retention times of prilocaine and lidocaine were 3.5 and 4.5 min, respectively. The calibration curves were created using standards of lidocaine with concentrations ranging from 0.097 to 200 µg/mL.

In vitro permeation profiles were generated by plotting the cumulative amount (Q, µg/cm^2^) and flux (J, µg/cm^2^/h) of lidocaine permeated versus time (h) [27]. The steady-state flux (Jss, µg/cm^2^/h) across the exposure area of the epidermis (A, cm^2^) was estimated from the apparent steady-state slope of the linear region in the plot of the cumulative amount versus time using Equation (2):

(2) $$Q=J_{SS}\cdot A\cdot \left(t-t_{lag}\right)$$

where t_lag_ (h) represents the lag time to reach the steady state of permeation [4]. The permeability coefficient was calculated with Fick’s law equation (Equation (3)):

(3) $$J_{SS}=K_{p}\cdot C_{v}$$

where K_p_ (cm^2^/h) is the permeability coefficient and C_v_ (µg/mL) is the drug concentration in the donor.

### 2.7. Statistical Analysis

The experimental data, including rheological properties and in vitro permeation parameters, was plotted and statistically analyzed using GraphPad Prism version 9.3.1 (GraphPad Software Inc., La Jolla, CA, USA) and Origin 2022b (OriginLab Corporation, Northampton, MA, USA). Data was expressed as the mean ± standard error where feasible. A one-way analysis of variance (ANOVA) was carried out to test differences at the 95% (*p* < 0.05) significance level between treatments.

## 3. Results

### 3.1. Evaporation Profile of O/W Lidocaine Cream

**Figure 1:**
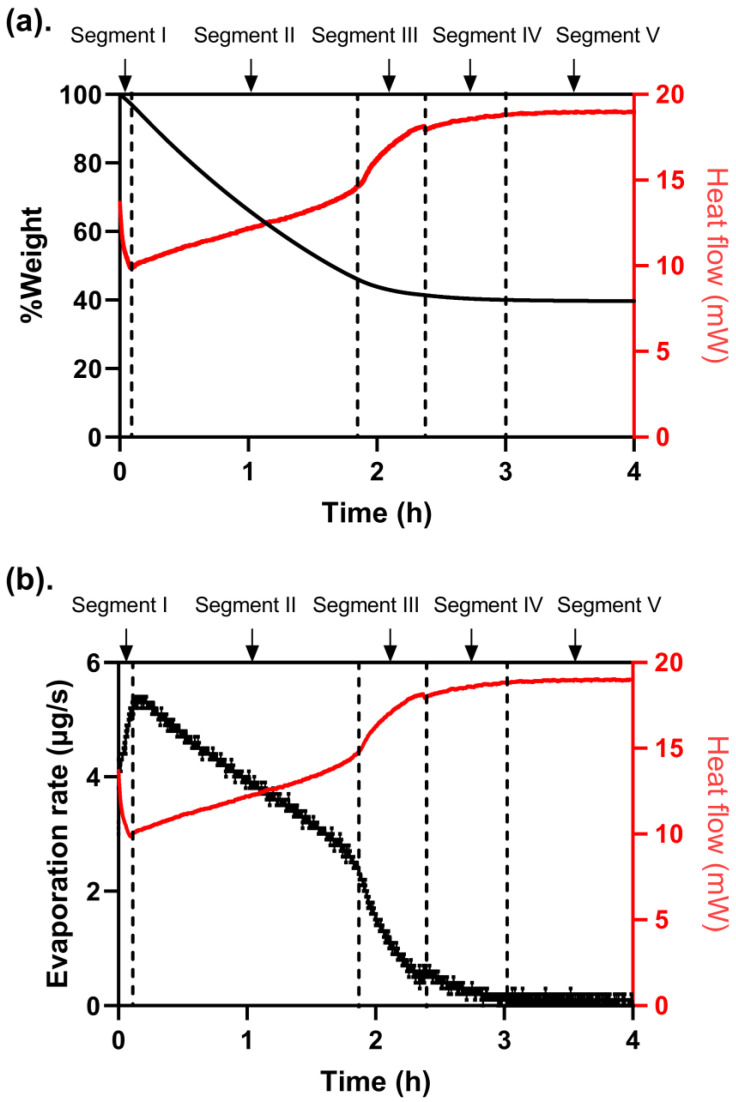
%Weight loss of O/W lidocaine cream F1 versus time (**a**) during the TGA and extrapolated evaporation rate (µg/s) versus time (**b**), with heat flow simultaneously measured by DSC plotted on the right *Y*-axis. Five segments were observed during the evaporation. Segment I: the evaporation of IPA; Segment II: the steady evaporative process of volatile solvents; Segment III: the crystallization of lidocaine; Segment IV: approaching the dissolution-crystallization balance; Segment V: the end of evaporation.

The DSC (heat flow variation) and TGA (%weight variation) curves, recorded with a sample of O/W lidocaine cream F1 subjected to the isothermal cycle performed under 32 °C from 0 to 4 h, are shown in Figure 1a. During the isothermal cycle, five segments were identified. According to the composition of the formulation and the exhibited evaporation profile in Figure 1b, the initial decline of heat flow in segment I (0–332 s) denoted the endothermic evaporation of IPA, while the subsequential exothermic process indicated the seeding and crystallization of lidocaine along with the evaporation of volatile solvents from the O/W system. After the steady increase of heat flow in segment II (332–6577 s), the surge of heat flow in segment III (6578–8526 s) suggested that drastic crystallization happened accompanied by a sharp decrease of evaporation rate till a dissolution-crystallization balance was reached at the end of segment IV (8527–10,763 s). The mass balance was achieved at the beginning of segment V (10,763–14,400 s), indicating non-volatile components accounted for 40.05% of the total weight of the sample.

### 3.2. Shear Flow Properties of O/W Lidocaine Cream and Modeling of “in Use” Apparent Viscosity

**Figure 2:**
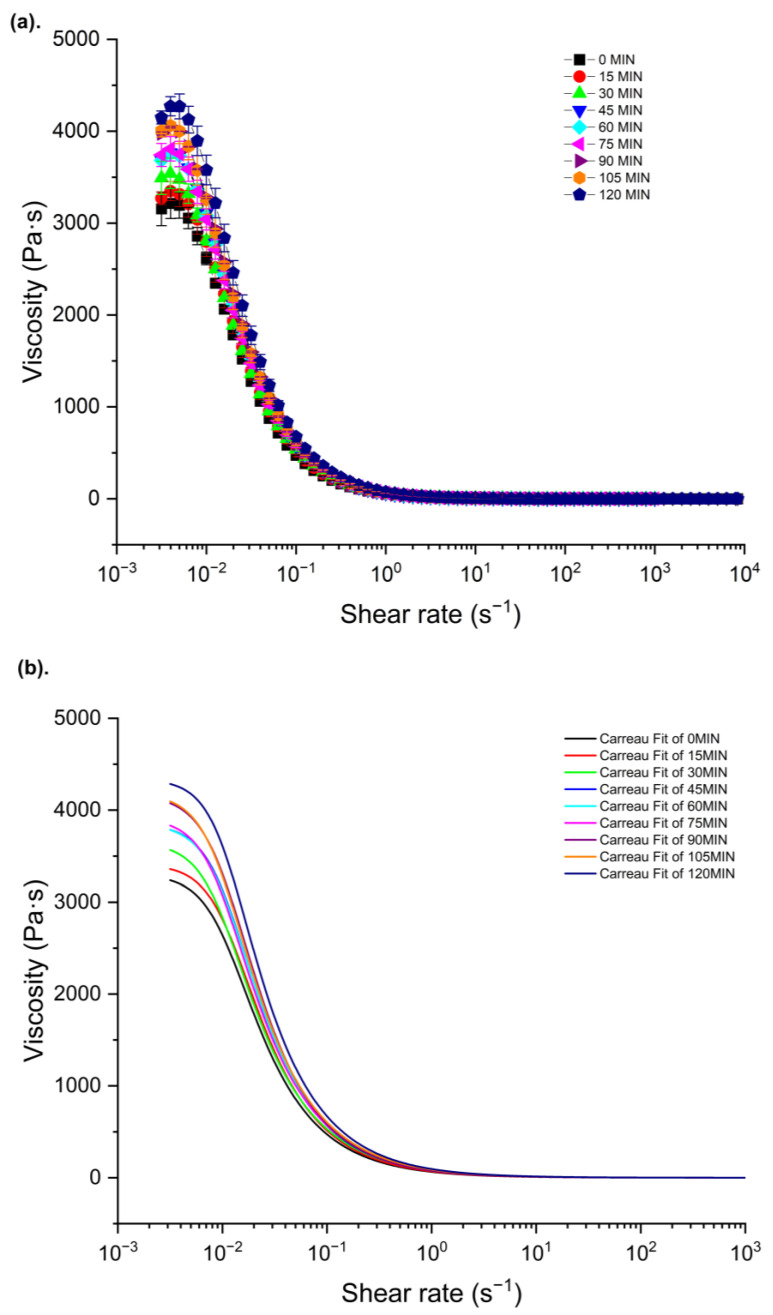
Experimental flow curves of O/W lidocaine cream F1 at different evaporative statuses under 32 °C (**a**) and their Carreau–Yasuda model-fitted curves (**b**). Data plotted as viscosity (Pa·s) versus shear rate (s^−1^) on a log scale.

The apparent viscosity values of O/W lidocaine cream F1 at different evaporative statuses under 32 °C are plotted in Figure 2a as a function of shear rate. Before the steep decrease from 0.004 s^−1^, the apparent viscosity remained flat between the first three measuring points (0.002 to 0.004 s^−1^), indicating a possible plateau with a low shear rate at near zero shear stress [28].With the further growth of the shear rate, a shear thinning behavior was observed for all samples till the infinite shear plateau (1 to 10,000 s^−1^) was reached, representing the disentanglement of carbopol polymer and aggregation of droplets under higher shear [29]. As expected, the apparent viscosity of cream samples rose with increasing evaporation time.

The experimental apparent viscosity data of lidocaine cream F1 were fitted to the Carreau–Yasuda model and generated 10 curves with R^2^ = 0.99 in Figure 2b, representing the flow behavior of the cream at different metamorphotic statuses under “in use” settings. Zero-shear viscosity ($\eta_{0}$), infinite-shear viscosity ($\eta_{\infty }$), and other parameters obtained by modeling are listed in Table 3. A solid alignment between experimental data and model outputs was achieved, as seen in the modest standard error. The increased values of the zero-shear viscosity from 3300 to 4334 Pa·s as a function of evaporation time indicated a high resistance to the movement of flow in the cream samples at a low shear rate. For all samples, the predicted values of infinite-shear viscosity were close to 0, suggesting the full disentanglement of microstructure under the extremely high shear rate [30,31,32,33]. The power indexes, n, which were smaller than 1, were consistent with the shear-thinning behavior of samples.

### 3.3. Impact of Metamorphosis and Carbopol Concentration on Viscosity

**Figure 3:**
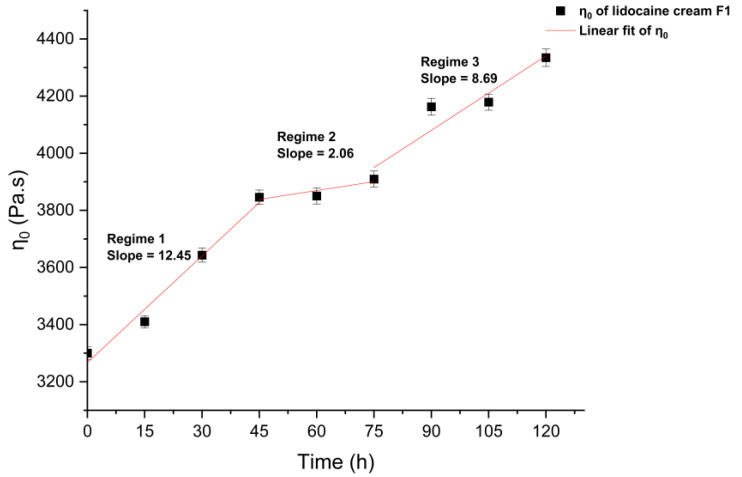
Evaporation/time dependence of the zero-shear viscosity ($\eta_{0}$) of O/W lidocaine cream F1. Data predicted by the Carreau–Yasuda model are adapted for linear curve fitting.

Figure 3 represents a plot of the zero-shear viscosity ($\eta_{0}$) versus the time of evaporation. Three distinct regimes were observed with slopes of 12.45, 2.06, and 8.69, suggesting different rates of increase in zero-shear viscosity as a result of losing volatile solvents during the metamorphosis of the formulation. The results showed that the rate of increase in zero-shear viscosity partially followed the trend of evaporation rate derived from the previous TGA, where the difference could be attributed to the difference in surface area for evaporation.

**Figure 4:**
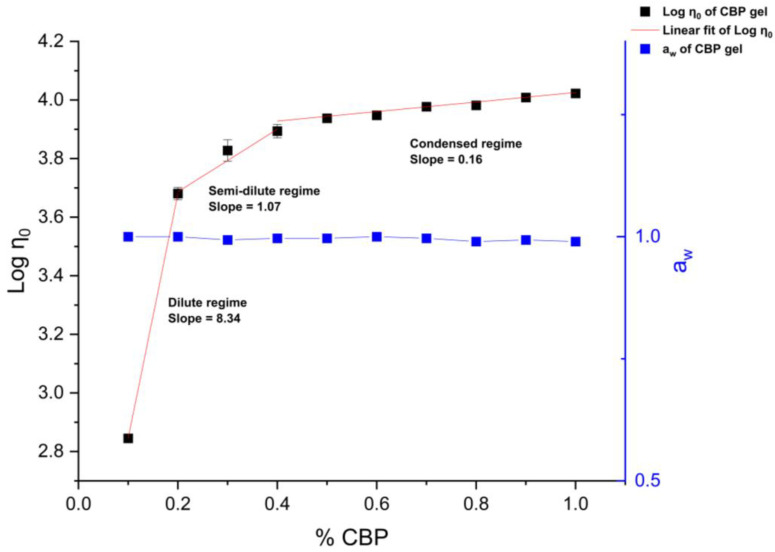
Concentration dependence of zero-shear viscosity ($\eta_{0}$) and water activity (a_w_) of CBP fluids. Data predicted by the Carreau–Yasuda model are adapted for linear curve fitting.

Considering carbopol as a non-volatile component in the formulation, its concentration increased with the incremental time of evaporation after application, and hence it was hypothesized that this caused the increase in viscosity. Cabropol was also used as a thickening agent to suspend droplets in the cream. Therefore, its contribution to increasing the viscosity was investigated by the prediction of zero-shear viscosity at 32 °C using the preceding modeling method. A logarithmical plot of zero-shear viscosity versus carbopol concentration in formulations is shown in Figure 4. Similarly, three regimes were identified, including a dilute regime with a slope of 8.34 at the low concentration range from 0 to 0.2%, a semi-dilute regime with a slope of 1.07 at the medium concentration range from 0.2 to 0.4%, and a condensed regime with a slope of 0.16 at the high concentration range from 0.4 to 1%. The increase in viscosity of carbopol fluids was consistent with the increasing time of evaporation. However, the water activity of all formulations remained stable at around 1, suggesting that most of the water is unbound in the polymeric system.

### 3.4. Viscoelastic Properties and Impacts of Metamorphosis on Oscillatory Rheogram

**Figure 5:**
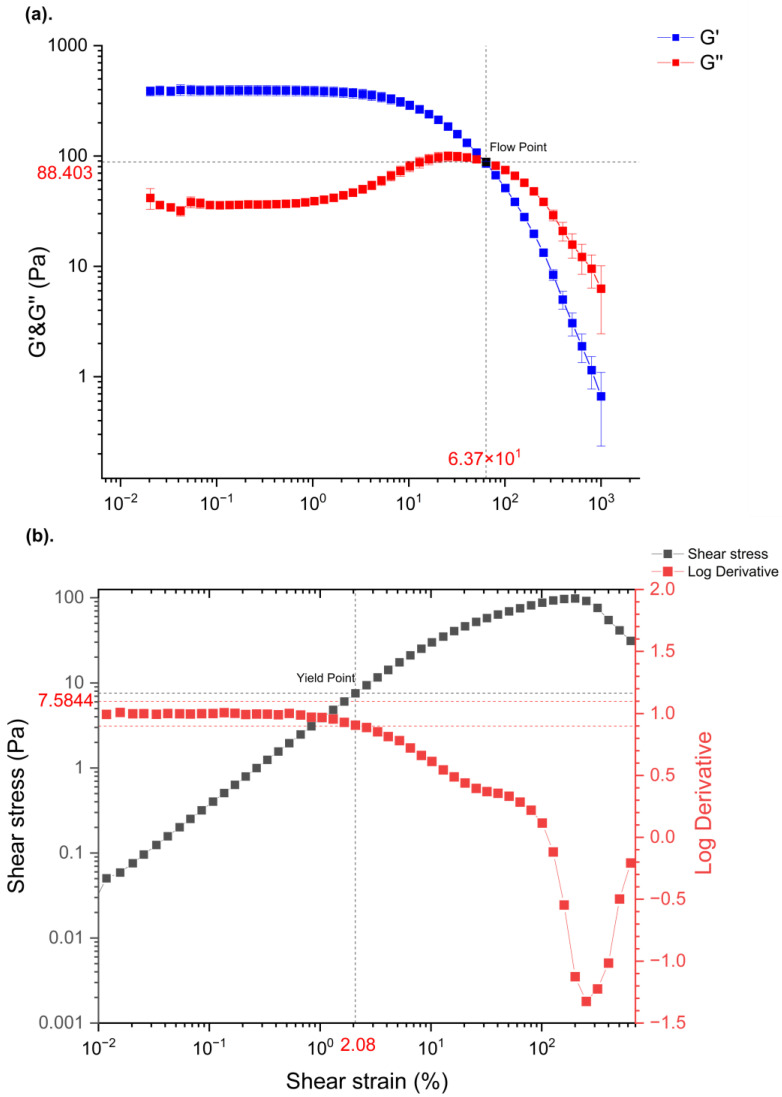
Elastic modulus (G′) and viscous modulus (G″) curves of (**a**) O/W lidocaine cream F1 samples at controlled shear strain, with black reference lines placed at the crossover of G′ and G″. Shear stress (τ) and its log derivative curves (**b**) as a function of shear strain, with red horizontal reference lines placed at a 0.1 offset of the log derivative.

The dynamic modulus and shear stress of the lidocaine cream F1 sample at 32 °C under controlled shear strain with oscillatory amplitude sweep are plotted in Figure 5. As shown in this Figure, a plateau of elastic modulus (G′; Figure 5a) and viscous modulus (G″; Figure 5b), i.e., LVR, was found before the decrease of G′ and increase of G″. G″ exceeded G′ after the flow point at 63.7% strain, suggesting elastic components, such as aggregated micelles of carbopol, had a predominant contribution to the microstructure in the formulation at low shear strain before the viscous fraction of the sample dominated the flow behavior at the high shear strain that can indicate the sol/gel transition time [34]. The LVR (from 0.01 to 2.08%) was determined by the log derivative of shear stress (τ) plotted in Figure 5b, where a 0.1 offset was considered the critical limit of the LVR. The yield point at the exit of the LVR, where a substantial deformation of the carbopol polymeric network occurred, was determined at 2.08% shear strain with 7.58 Pa of shear stress.

**Figure 6:**
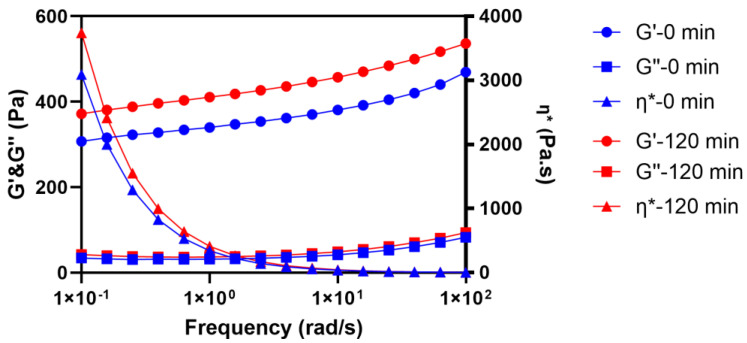
Frequency sweep of O/W lidocaine cream F1 samples at 0 and 120 min as a function of logarithmically controlled frequency. G′: elastic modulus; G″: viscous modulus; η*: complex viscosity.

To understand the impacts of metamorphosis on the viscoelastic properties of the formulation under “in use” conditions, a frequency sweep was performed using 1% strain at 32 °C. The rheograms of O/W lidocaine cream F1 samples within the LVR at 0 and 120 min are depicted in Figure 6. The trace of G′ was constantly above G″, and both moduli remained stable despite the incremental frequency while the complex viscosity (η*) gradually decreased, indicating a gel-like profile of the samples. However, an enlarged gap between G′ and G″ could be observed after 2 h of evaporation due to the parallel increase of G′ over the range of frequency from 0.1 to 100 rads, suggesting a denser 3D matrix of carbopol was generated as a result of metamorphosis [35].

### 3.5. In Vitro Skin Permeation Profiles of Lidocaine

**Figure 7:**
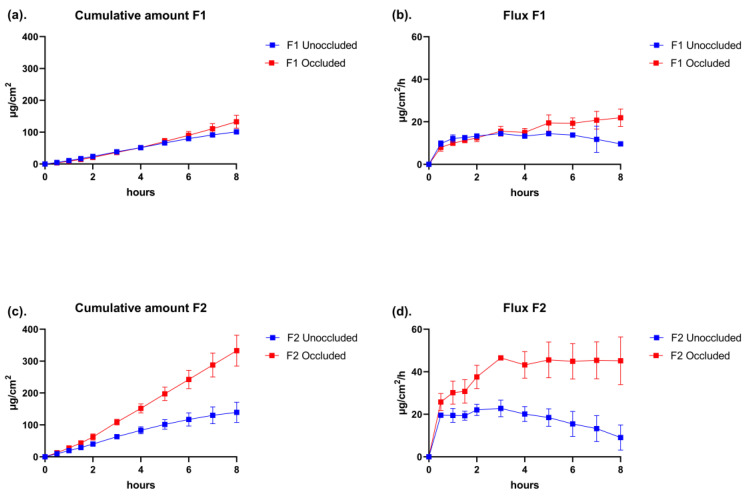
In vitro permeation profiles of lidocaine in O/W lidocaine cream F1 and F2 with or without occlusion. (**a**) Cumulative amount of lidocaine in F1 under unoccluded and occluded conditions. (**b**) flux of lidocaine in F1 under unoccluded and occluded conditions. (**c**) Cumulative amount of lidocaine in F2 under unoccluded and occluded conditions. (**d**) flux of lidocaine in F2 under unoccluded and occluded conditions. Data are presented as Mean ± SEM, n = 3.

To investigate the skin permeation profile of lidocaine under the “in use” condition and its relationship to the metamorphosis of formulation, an IVPT study with cream formulations listed in Table 1 was performed. The flux and cumulative amount are shown in Figure 7. A good linear region of the cumulative amount was achieved for all formulations under both unoccluded and occluded conditions, and a higher penetration amount of lidocaine was found in occluded cells, suggesting the permeation enhancement caused by occlusion. In unoccluded cells, the steady state of penetration for both formulations ended after 4 h of the experiment since their flux started to decrease, which could be attributed to the increase in viscosity. Another possible reason is the crystallization of lidocaine with the evaporation of volatile solvents. As APIs in crystalline form cannot permeate through the skin barrier, the bioavailability of lidocaine in the formulation is thereby reduced. The flux of lidocaine in occluded cells remained stable until the end of the study, suggesting the drug in both formulations was not depleted. Compared to F1, a higher flux and cumulative amount of F2 were found, which was considered to be caused by the higher concentration and thermodynamic activity of lidocaine in the formulation.

The skin permeation parameters were calculated using Equations (2) and (3) and listed in Table 4. Lidocaine in unoccluded diffusion cells dosed with both formulations exhibited a lower J_ss_ and lower K_p_, suggesting a retardant effect on skin penetration due to evaporation. The increase in K_p_ with the increase in lidocaine concentration in the donor is consistent with the penetration enhancement illustrated in Figure 7. The steady-state flux was reached earlier in unoccluded diffusion cells as a reduced lag time (t_lag_) was observed.

## 4. Discussion

**Figure 8:**
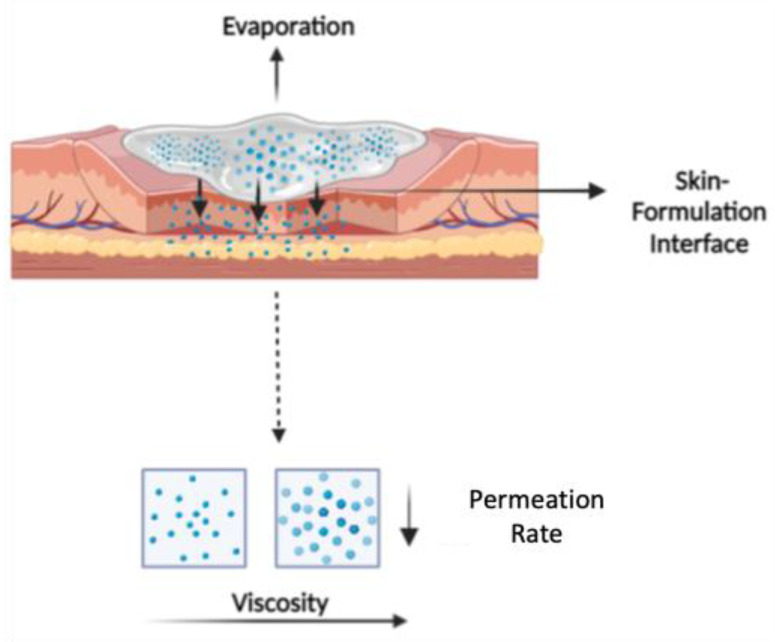
A graphical illustration of the interplay between the applied formulation and skin. The viscosity of topical semisolid products increased with the evaporation of volatile solvents, resulting in a more compact product microstructure at the skin-formulation interface. Thus, the permeation rate of APIs from the formulation was significantly reduced, indicating the product’s lower permeability and overall therapeutic efficacy.

In the case of complex preparations, such as topical semisolid products, the metamorphosis of formulation, such as evaporation and crystallization, can significantly impact the bioavailability of drug products. The evaporation rate of different marketed topical products with various dosage forms, such as solution, lotion, gel, cream, and ointment, can differ based on the concentrations of volatile excipients (e.g., water, ethanol, and propylene glycol) used in the structure of these products [36,37,38,39]. For example, a gel, compared to an ointment, evaporates more rapidly owing to the higher content of volatiles, such as water and alcohol, in the gel structure [37]. Additionally, it has been demonstrated that maximizing the saturation percentage of APIs (i.e., thermodynamic activity) has a crucial role in optimizing the skin delivery of topical formulations [1]. Drying up the topical products, such as gels containing volatile vehicles (e.g., water and ethanol), after topical application causes a thermodynamically unstable supersaturated system and, subsequently, its crystallization. This results in a decrease in the skin permeation of the product [5]. Depending on the dosage form, a range of CQAs, such as particle size, globule size, rheological properties, and thermodynamic activity, can be altered when the topical products encounter the metamorphosis, leading to specific failure modes [13]. Therefore, in this study, the role of rheological properties, such as viscosity and viscoelastic behavior, in drug permeation under the “in use” condition was investigated. In this work, experimental conditions were not synchronized to allow for evaporation measurements up to a 4 h duration in DSC/TGA, which provided a sensitive microbalance and control on environmental conditions and narrow gap rheological measurement to study the evaporation of formulation and metamorphotic impacts on drug permeation under true in-use conditions of a finite dose (10 mg/cm^2^), according to OECD guidelines. As illustrated in Figure 8, the API-excipient mixture is trapped in the network of crosslinked polymer chains in the hydrophilic gel phase, and the polymeric content rises due to the simultaneous evaporation and absorption of drug vehicles into the skin, resulting in a condensed carbopol gel network that has a retardant effect on the permeability of API.

TGA and DSC results provide a unique opportunity to decipher the dynamic metamorphosis of lidocaine cream from primary to tertiary formulation, where a series of metamorphotic events, such as evaporation and crystallization, occur and eventually lead to a change in rheological behavior and drug permeability. The evaporation rate determined by isothermal TGA, which showed five prominent segments at skin temperature (Figure 1), was linked to the simultaneously measured heat flow, suggesting that the evaporation kinetics of cream were likely related to the crystallization of lidocaine in the colloidal matrix of carbopol. In one study [25], similar five segments for the evaporation of water were observed in colloidal unimolecular polymer systems, where the alteration of surface tension and viscosity were attributed to the change in evaporation rate during the isothermal process [25]. For further differentiation of the evaporation rate of water and other cosolvents, the latest developed method to measure the loss of water using a customized evaporimeter directly and to compare the water loss to the weight loss of other volatiles has been disclosed in the recent literature [40].

The measured apparent viscosity data of lidocaine cream under the “in use” condition were aligned with the Carreau–Yasuda model, thus giving great potential to predict the zero-shear viscosity of the cream as a function of evaporation time. Three regimes with different slopes reported in Figure 4 reflect the rearrangement of microstructure in the complex mixture system during the metamorphosis. The growth of zero-shear viscosity with time is likely to indicate that a higher activation energy, which is strongly dependent on the interaction of polymer chains, is required for the movement of molecules, which, in turn, can possibly result in a lower diffusivity [41,42]. In line with the flow behavior data of topical formulations with varying concentrations of excipients from Li et al., the prediction of zero-shear viscosity of carbopol fluids, which is shown in Figure 5, further supports this, as three similar regimes can be easily identified [34]. The dynamic rheological properties were assessed by amplitude sweep and frequency sweep to evaluate the viscoelasticity of the lidocaine cream formulation. The elastic modulus and viscous modulus plotted in the oscillatory rheography (Figure 5) and derived parameters, including flow point and yield point, were consistent with the previous rheological characterization of commercially available creams [43]. As Figure 5a shows, G″ exceeded G′ after the flow point at 63.7% strain, which could indicate the sol/gel transition time [34]. The rheogram of the cream plotted in Figure 6 exhibited a gel-like profile since the trace of G′ was constantly above G″, indicating that the sample formed a continuous network structure, resulting in a strong gel [34]. This implied that the elastic components, such as anionic carbopol clusters, are predominant structural entities in situ after the formulation is applied to the skin [44]. The increased elastic modulus of samples after 2 h of evaporation also suggested the condensing of carbopol in the formulation as a result of metamorphosis.

By integrating the predicted zero-shear viscosity, the significant influence of metamorphosis on the permeation profile of lidocaine cream formulations, i.e., the retardant effect on skin penetration, was successfully captured in the IVPT study, where the evaporation condition was manipulated with the occlusion of diffusion cells. Cross et al. [45] studied the penetration of oxybenzone emulsions containing the thickening agent carbomer 940 (from 0% to 0.5%) under both infinite dose (static) and finite dose (in-use) conditions across human skin [45]. Both the results of the current and Cross et al. studies verified that the drug flux was inversely proportional to the viscosity of the formulation under the “in use” condition. Additionally, the loss of volatile cosolvents due to metamorphosis, especially IPA in this case, evolved the formulation mixture into a thermodynamically unstable system, resulting in the spontaneous crystallization of lidocaine [46,47]. Hence, bioavailability was reduced. Mostly, the evaporation rate of volatile solvents is faster than the permeation rate of APIs and excipients, as these molecules need to go through the hydrophobic extracellular spaces between keratinocytes filled with lipid lamellar in the stratum corneum to get into the skin. Similarly, literature also shows that the total mass loss due to evaporation is higher than the measured transepidermal water loss on skin applied with the formulation [40]. The real-time quantitative analysis for this dynamic process was observed by Belsey et al. using stimulative Raman scattering (SRS) microscopy, and they mapped the topography of Ibuprofen-d3 crystal on the surface and in the multiple layers of the skin to reveal the metamorphosis of topical formulations [48].

In this work, we witnessed the metamorphosis of topical semisolid formulation caused by evaporation and acknowledged its implication on the rate and extent of percutaneous drug permeation, which was less significant from past IVPT infinite dose studies. Although the metamorphosis has been taken into consideration for QTPP by the evolving pharmaceutical industry, this issue is addressed here for the “in use” condition. Overall, such a relationship observed between evaporation and changes in viscosity and drug permeability can provide valuable insights into the metamorphosis of topical semisolid products under the “in use” condition that can lead to specific failure modes of the product, such as crystallization, for instance. The stepwise workflow proposed in this work, including TGA, rheological characterization, and IVPT studies, could be adapted for other topical semisolid formulations to understand the impacts of metamorphosis on a case-by-case basis for different APIs and physicochemical properties of formulations. By further integrating physiologically based pharmacokinetic modeling, the failure modes can be predicted and mitigated for the development of similar or different Q3 products.

## 5. Conclusions

As far as we are aware, this is the first work presenting the rheological change of topical semisolid formulation simultaneously with the evaporation of volatile solvent that led to a concurrent reduction of API’s skin permeability. It was found that with the evaporation of volatile components from lidocaine cream, the permeability of lidocaine decreased while the viscosity and elastic modulus of the cream, as a result of the aggregation of carbopol micelles and crystallization of lidocaine after application, increased. Along with the physical and structural characterization of topical semisolid products, the potential failure modes for similar or different Q3 products will be predictable by further understanding of the metamorphotic events and changes in CQAs. This work and the revealed underlying mechanism provide mathematical modelers with the necessary background to build complex models that incorporate evaporation, viscosity, and drug permeation in the simulation once at a time.

## Figures and Tables

**Table 1:** Compositions of lidocaine O/W cream.

Ingredients	F1		F2	
	%*w*/*w*	g	%*w*/*w*	g
Lidocaine	2.5	5	5	10
Isopropyl alcohol	15	30	15	30
Isopropyl myristate	5	10	-	-
Sodium lauryl sulfate	0.75	1.5	1.5	3
Carbopol 980	0.75	1.5	0.75	1.5
Water q.s.	100	152	100	155.5
Total		200		200

**Table 2:** Compositions of carbopol fluids.

Formulation	Ingredients		
	% Carbopol 980 (*w*/*w*)	% Sodium Hydroxide (*w*/*w*)	% Water (*w*/*w*)
CBP01	0.1	0.23 q.s.	99.67
CBP02	0.2	0.46 q.s.	99.34
CBP03	0.3	0.69 q.s.	99.01
CBP04	0.4	0.92 q.s.	98.68
CBP05	0.5	1.15 q.s.	98.35
CBP06	0.6	1.38 q.s.	98.02
CBP07	0.7	1.61 q.s.	97.69
CBP08	0.8	1.84 q.s.	97.36
CBP09	0.9	2.07 q.s.	97.03
CBP10	1.0	2.30 q.s.	96.70

**Table 3:** Carreau–Yasuda model parameters for O/W lidocaine cream F1 based on apparent viscosity measurement $\eta_{0}$: zero-shear viscosity (Pa·s); $\eta_{\infty }$: infinite shear viscosity (Pa·s); λ: time constant; a: transition control factor; n: power index.

Time (min)	$\eta_{0}$ (Pa·s)	$\eta_{\infty }$ (Pa·s)	λ (s)	a	n	R^2^
0	3300.17 ± 22.65	0 ± 3.74	93.12 ± 2.51	2.39 ± 0.13	0.13 ± 0.02	0.99
15	3410.21 ± 21.03	0 ± 3.89	86.80 ± 2.30	2.43 ± 0.12	0.13 ± 0.02	0.99
30	3643.47 ± 24.69	0 ± 3.78	104.98 ± 2.40	2.46 ± 0.13	0.18 ± 0.01	0.99
45	3846.12 ± 25.01	0 ± 4.45	91.97 ± 2.41	2.48 ± 0.13	0.14 ± 0.02	0.99
60	3850.47 ± 28.16	0 ± 4.85	93.39 ± 2.67	2.43 ± 0.14	0.15 ± 0.02	0.99
75	3909.73 ± 27.79	0 ± 4.39	101.74 ± 2.54	2.45 ± 0.13	0.17 ± 0.02	0.99
90	4162.96 ± 29.10	0 ± 4.60	91.85 ± 2.56	2.31± 0.12	0.12 ± 0.02	0.99
105	4178.92 ± 28.36	0 ± 4.47	101.29 ± 2.43	2.45 ± 0.13	0.17 ± 0.01	0.99
120	4334.91 ± 30.89	0 ± 6.15	90.59 ± 2.67	2.64 ± 0.17	0.15 ± 0.02	0.99

**Table 4:** Skin permeation parameters of lidocaine in O/W lidocaine cream F1. C_V_: concentration of donor; J_ss_: steady-state flux; t_lag_: lag time; K_p_: permeability coefficient. Data are expressed as mean ± SEM, n = 3. * Data are significantly different (*p* < 0.05).

Formulation	Occlusion	C_V_	*J_ss_	*t_lag_	*K_p_
		(mg/mL)	(µg/cm^2^/h)	(h)	(cm^2^/h)
F1	−	25	12.37 ± 0.68	0.28 ± 0.2	4.95 × 10^−4^ ± 2.72 × 10^−5^
F1	+	25	18.3 ± 5.34	1.61 ± 1.92	7.32 × 10^−4^ ± 2.14 × 10^−4^
F2	−	50	20 ± 1.67	0.21 ± 0.16	4.00 × 10^−4^ ± 3.34 × 10^−5^
F2	+	50	40.03 ± 7.93	0.64 ± 1.22	8.01 × 10^−3^ ± 1.59 × 10^−3^

## Data Availability

Not applicable.

## References

[1] Mohammed Y., Holmes A., Kwok P.C.L., Kumeria T., Namjoshi S., Imran M., Matteucci L., Ali M., Tai W., Benson H.A. (2022). Advances and future perspectives in epithelial drug delivery. Adv. Drug Deliv. Rev..

[2] Barrett C.W., Hadgraft J.W., Sarkany I. (1964). The influence of vehicles on skin penetration. J. Pharm. Pharmacol..

[3] Barry B.W. (1987). Mode of action of penetration enhancers in human skin. J. Control. Release.

[4] Roberts M.S., Cheruvu H.S., Mangion S.E., Alinaghi A., Benson H.A., Mohammed Y., Holmes A., van der Hoek J., Pastore M., Grice J.E. (2021). Topical drug delivery: History, percutaneous absorption, and product development. Adv. Drug Deliv. Rev..

[5] Jin X., Imran M., Mohammed Y. (2022). Topical Semisolid Products—Understanding the Impact of Metamorphosis on Skin Penetration and Physicochemical Properties. Pharmaceutics.

[6] Hummer J., Birngruber T., Sinner F., Page L., Toner F., Roper C.S., Moore D.J., Baker M.B., Boncheva Bettex M. (2022). Optimization of topical formulations using a combination of in vitro methods to quantify the transdermal passive diffusion of drugs. Int. J. Pharm..

[7] Miranda M., Cardoso C., Vitorino C. (2020). Quality and equivalence of topical products: A critical appraisal. Eur. J. Pharm. Sci..

[8] Roberts M.S., Cross S.E. (1999). Percutaneous absorption of topically applied NSAIDS and other compounds: Role of solute properties, skin physiology and delivery systems. Inflammopharmacology.

[9] Yang D., Liu C., Ding D., Quan P., Fang L. (2021). The molecular design of drug-ionic liquids for transdermal drug delivery: Mechanistic study of counterions structure on complex formation and skin permeation. Int. J. Pharm..

[10] Osborne D. (2016). Impact of Quality by Design on Topical Product Excipient Suppliers, Part I: A Drug Manufacturer’s Perspective. Pharm. Technol..

[11] Sivaraman A., Banga A. (2015). Quality by design approaches for topical dermatological dosage forms. Res. Rep. Transdermal Drug Deliv..

[12] Simões A., Veiga F., Vitorino C. (2020). Progressing Towards the Sustainable Development of Cream Formulations. Pharmaceutics.

[13] Jung N., Namjoshi S., Mohammed Y., Grice J.E., Benson H.A.E., Raney S.G., Roberts M.S., Windbergs M. (2022). Application of Confocal Raman Microscopy for the Characterization of Topical Semisolid Formulations and their Penetration into Human Skin Ex Vivo. Pharm. Res..

[14] Namjoshi S., Dabbaghi M., Roberts M.S., Grice J.E., Mohammed Y. (2020). Quality by Design: Development of the Quality Target Product Profile (QTPP) for Semisolid Topical Products. Pharmaceutics.

[15] Wu K., Yeoh T., Hsieh Y.-L., Osborne D.W., Langley N., Michniak-Kohn B., Osborne D.W. (2019). Quality Assessment of API in Semisolid Topical Drug Products. The Role of Microstructure in Topical Drug Product Development.

[16] Surber C., Knie U. (2018). Metamorphosis of vehicles: Mechanisms and opportunities. pH Ski. Issues Chall..

[17] Langley N., Michniak B.B., Osborne D.W. (2019). The Role of Microstructure in Topical Drug Product Development.

[18] Kamal N.S., Krishnaiah Y.S.R., Xu X., Zidan A.S., Raney S., Cruz C.N., Ashraf M. (2020). Identification of critical formulation parameters affecting the in vitro release, permeation, and rheological properties of the acyclovir topical cream. Int. J. Pharm..

[19] Haq A., Chandler M., Michniak-Kohn B. (2020). Solubility-physicochemical-thermodynamic theory of penetration enhancer mechanism of action. Int. J. Pharm..

[20] Coldman M., Poulsen B., Higuchi T. (1969). Enhancement of percutaneous absorption by the use of volatile: Nonvolatile systems as vehicles. J. Pharm. Sci..

[21] Chia-Ming C., Flynn G.L., Weiner N.D., Szpunar G.J. (1989). Bioavailability assessment of topical delivery systems: Effect of vehicle evaporation upon in vitro delivery of minoxidil from solution formulations. Int. J. Pharm..

[22] Cui Y., Xu S., Wu S., Du S., Cao Y., Chen Y., Liu L., Dong W., Gong J. (2017). Temperature and solvent dependent thermodynamic behavior of tetrabromobisphenol A. J. Mol. Liq..

[23] Arora S., Clarke J., Tsakalozou E., Ghosh P., Alam K., Grice J.E., Roberts M.S., Jamei M., Polak S. (2022). Mechanistic modeling of in vitro skin permeation and extrapolation to in vivo for topically applied metronidazole drug products using a physiologically based pharmacokinetic model. Mol. Pharm..

[24] Kang L., Jun H.W. (2003). Formulation and Efficacy Studies of New Topical Anesthetic Creams. Drug Dev. Ind. Pharm..

[25] Geng P., Zore A., Van De Mark M.R. (2020). Investigation of the Evaporation Rate of Water from Colloidal Unimolecular Polymer (CUP) Systems by Isothermal TGA. Polymers.

[26] Kligman A.M., Christophers E. (1963). Preparation of isolated sheets of human stratum corneum. Arch. Dermatol..

[27] Seo J.-E., Kim S., Kim B.-H. (2017). In vitro skin absorption tests of three types of parabens using a Franz diffusion cell. J. Expo. Sci. Environ. Epidemiol..

[28] LEVER M.J. (2005). Mass transport processes in artificial organs. Biomaterials, Artificial Organs and Tissue Engineering.

[29] Ching S.H., Bansal N., Bhandari B. (2016). Rheology of emulsion-filled alginate microgel suspensions. Food Res. Int..

[30] Park E.-K., Song K.-W. (2010). Rheological evaluation of petroleum jelly as a base material in ointment and cream formulations: Steady shear flow behavior. Arch. Pharmacal Res..

[31] Barthelmes G., Pratsinis S.E., Buggisch H. (2003). Particle size distributions and viscosity of suspensions undergoing shear-induced coagulation and fragmentation. Chem. Eng. Sci..

[32] Park E.-K., Song K.-W. (2010). Rheological evaluation of petroleum jelly as a base material in ointment and cream formulations with respect to rubbing onto the human body. Arch. Pharm. Res..

[33] Park E.-K., Song K.-W. (2011). Rheological Evaluation of Petroleum Jelly as a Base Material in Ointment and Cream Formulations: Linear Viscoelastic Behavior. J. Pharm. Investig..

[34] Li C., Liu C., Liu J., Fang L. (2011). Correlation between rheological properties, in vitro release, and percutaneous permeation of tetrahydropalmatine. Aaps Pharmscitech.

[35] Brady J., Dürig T., Lee P., Li J.-X. (2017). Polymer properties and characterization. Developing Solid Oral Dosage Forms.

[36] Buhse L., Kolinski R., Westenberger B., Wokovich A., Spencer J., Chen C.W., Turujman S., Gautam-Basak M., Kang G.J., Kibbe A. (2005). Topical drug classification. Int. J. Pharm..

[37] Hunter A.M., Grigson C., Wade A. (2018). Influence of topically applied menthol cooling gel on soft tissue thermodynamics and arterial and cutaneous blood flow at rest. Int. J. Sport. Phys. Ther..

[38] Chen C.-P., Chen C.-C., Huang C.-W., Chang Y.-C. (2018). Evaluating Molecular Properties Involved in Transport of Small Molecules in Stratum Corneum: A Quantitative Structure-Activity Relationship for Skin Permeability. Molecules.

[39] Shin S.H., Rantou E., Raney S.G., Ghosh P., Hassan H., Stinchcomb A. (2020). Cutaneous Pharmacokinetics of Acyclovir Cream 5% Products: Evaluating Bioequivalence with an In Vitro Permeation Test and an Adaptation of Scaled Average Bioequivalence. Pharm. Res..

[40] Mohammed Y., Namjoshi S., Telaprolu K., Crowe A., Jung N., Grice J., Windbergs M., Benson H., Raney S.G., Roberts M.S. A Novel Method to Selectively Differentiate between the Loss of Water and Other Volatiles from Topical Semisolid Products. Proceedings of the 2017 Controlled Release Society Annual Meeting.

[41] Xu L., Xu G., Liu T., Chen Y., Gong H. (2013). The comparison of rheological properties of aqueous welan gum and xanthan gum solutions. Carbohydr. Polym..

[42] Varges P.R., Costa C.M., Fonseca B.S., Naccache M.F., De Souza Mendes P.R. (2019). Rheological Characterization of Carbopol^®^ Dispersions in Water and in Water/Glycerol Solutions. Fluids.

[43] Dabbaghi M., Namjoshi S., Panchal B., Grice J.E., Prakash S., Roberts M.S., Mohammed Y. (2021). Viscoelastic and deformation characteristics of structurally different commercial topical systems. Pharmaceutics.

[44] Morris E., Gothard M., Hember M., Manning C., Robinson G. (1996). Conformational and rheological transitions of welan, rhamsan and acylated gellan. Carbohydr. Polym..

[45] Cross S.E., Roberts M.S., Jiang R., Benson H.A. (2001). Can increasing the viscosity of formulations be used to reduce the human skin penetration of the sunscreen oxybenzone?. J. Investig. Dermatol..

[46] Welin-Berger K., Neelissen J.A.M., Bergenståhl B. (2001). The effect of rheological behaviour of a topical anaesthetic formulation on the release and permeation rates of the active compound. Eur. J. Pharm. Sci..

[47] Hadgraft J., Lane M.E. (2016). Drug crystallization—Implications for topical and transdermal delivery. Expert Opin. Drug Deliv..

[48] Belsey N.A., Garrett N.L., Contreras-Rojas L.R., Pickup-Gerlaugh A.J., Price G.J., Moger J., Guy R.H. (2014). Evaluation of drug delivery to intact and porated skin by coherent Raman scattering and fluorescence microscopies. J. Control. Release.

